# A Novel Dicyanoisophorone-Based Ratiometric Fluorescent Probe for Selective Detection of Cysteine and Its Bioimaging Application in Living Cells

**DOI:** 10.3390/molecules23020475

**Published:** 2018-02-22

**Authors:** Hengrui Zhang, Nan Qin, Zhijie Fang

**Affiliations:** School of Chemical Engineering, Nanjing University of Science & Technology, 200 Xiao Ling Wei, Nanjing 210094, China; zhrlab201@163.com (H.Z.); qnlab408@126.com (N.Q.)

**Keywords:** fluorescent probe, dicyanoisophorone, cysteine, ratiometric

## Abstract

A selective and ratiometric turn-on fluorescent probe was designed and synthesized by using a novel dicyanoisophorone-based derivative and acrylate moiety. The probe displayed high stability and good selectivity to cysteine (Cys) over homocysteine (Hcy) and glutathione (GSH). It also exhibited rapid response to Cys within 180 s. Most importantly, the fluorescence intensity ratio at 590 and 525 nm (I_590_/I_525_) was linearly dependent on the Cys concentration in the range from 0 to 40 μM and the detection limit calculated to be 0.48 μM. This probe was also applied for bioimaging of intracellular Cys in living HeLa cells with low cytotoxicity.

## 1. Introduction

As an important kind of biological thiols, cysteine (Cys) plays a crucial role in many significant cellular functions [1,2,3]. Abnormal level of Cys is closely related to many diseases, including slow growth, skin lesions and weakness, loss of muscle and fat, hair depigmentation, edema, metabolic disorders and so on [4,5,6]. Therefore, much effort has been given to develop selective and sensitive assays for the detection of Cys in biological samples [7,8,9]. Fluorescent chemosensors are highly desirable in practical applications, based on their merits of convenience, high sensitivity and rapid performance [10,11,12,13,14].

Ideal fluorescent probes should possess good selectivity and lower interference. Owing to the similarities among molecular backbone and reaction of Cys and homocysteine (Hcy)/glutathione (GSH), the key challenge of fluorescence bioimaging for Cys is to detect Cys specifically over other biologically relevant thiols [15,16,17]. Various groups have been employed as the recognition unit, including thiophenols [18], maleic anhydride [19], acrylate [20,21]. Among these, the conjugate addition-cyclization of Cys to acrylate moiety was considered as one of the most efficient strategy to design Cys-selective fluorescent probes. Another challenge is that fluorescence intensity-based measurements tend to be affected by sample environment, instrumental efficiency and probe molecule concentration [22,23,24]. These limits can be addressed by ratiometric fluorescent response, which is more suitable for use in biosystems. However, sofar only a few Cys selective ratiometric fluorescent have been reported [25,26,27,28].

In recent years, many dicyanoisophorone-based probes have rapidly been developed due to its good fluorescence properties and simplicity [29,30,31,32,33]. Since our group has synthesized several fluorescent probes [34,35,36], as a continuation of our work in this field, we herein designed and synthesized a novel dicyanoisophorone-based fluorescent probe **1** for detecting Cys accurately. The probe showed high selectivity and sensitivity for Cys over various analytes including Hcy and GSH. Moreover, the probe displayed ratiometric detection of Cys and was applied to fluorescent imaging of intracellular Cys in living cells.

## 2. Results and Discussion

### 2.1. Synthesis and Characterization of Probe ***1***

**Scheme 1:**
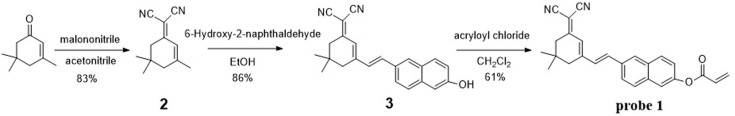
Synthesis of probe **1**.

Probe **1** was synthesized by using esterification reaction of compound **3** with acryloyl chloride in 83% yield. The important intermediate compound **3** was prepared from compound **2** and 6-hydroxy-2-naphthaldehyde by a condensation reaction in 86% yield. Isophorone was used as the raw material to synthesis compound **2** in 61% yield (Scheme 1). Moreover, Compound **3** had not been reported before. The chemical structure of probe **1** was fully identified by ^1^H-NMR, ^13^C-NMR and HRMS. Detailed synthetic procedures and structure characterizations were given in the experimental section and supporting information.

### 2.2. Rapid and Ratiometric Optical Response of Probe ***1*** to Cys

**Figure 1:**
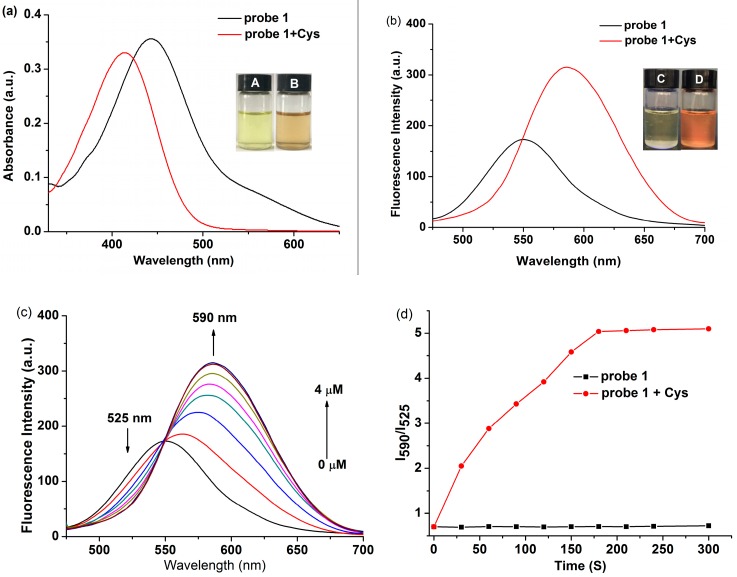
(**a**) UV-vis spectra changes and (**b**) fluorescence spectra changes of probe **1** (10 μM) before and after incubation with Cys (50 μM) in PBS buffer (10 mM, pH 7.4) at 37 °C for 20 min. Fluorescence and color photographs of probe **1** in the absence (**a**,**c**) and presence (**b**,**d**) of Cys are inserted; (**c**) Fluorescence spectra of **1** (10 μM) in the presence of various concentrations of Cys from 0 to 40 μM; (**d**) Time-dependent fluorescence ratio changes of probe **1** in the presence (red) and absence (black) of Cys (50 μM). Data was recorded every 30 s. For fluorescence measurement, λ_ex_ = 410 nm, d_ex_ = 3 nm, d_em_ = 5 nm.

The response of probe **1** for Cys was studied in PBS/DMSO solution (1:1, *v*/*v*, pH = 7.4, 10 mM). When 50 μM of Cys was added to the solution containing probe **1** (10 μM), the color changed from pale yellow to orange rapidly. What’s more, probe **1** exhibited a maximum absorption peak at 410 nm. Upon the addition of Cys toprobe **1**, the absorption peak at 410 nm disappeared, accompanied by the emergence of a new absorption peak at 445 nm (Figure 1a). In the fluorescence spectra, it can be seen that probe **1** showeda weak fluorescence under an excitation wavelength at 445 nm. From the fluorescence spectra recorded upon exciation at 445, it can be seen that the initial emission maximum of probe **1** changes from approx. 550 nm to 590 nm upon the addition of cysteine, accompanied with a naked-eye-visible appearance of the orange colour (Figure 1b). One of the possible explanations for this fluorescence change might be a restoration of the internal charge transfer (ICT) process upon the removal of the acrylate protecting group from the phenolate moiety(this is just a speculation, and has not been investigated in detail). To ensure that the observable changes in the fluorescent properties were not influenced by the insolubility/aggregation effects, the linearity of absorbance and fluorescence against probe **1** concentration (without the addition of Cys) were verified in the concentration range of 1–100 μM (Figure S1).

Then the fluorescence properties of probe **1** with different concentrations of Cys (0–40 μM) were investigated. The fluorescence intensity at 525 nm gradually decreased, and the emission at 590 nm increased dramatically with increasing concentrations of Cys until reaching saturation (Figure 1c). The fluorescence intensity ratio at 590 and 525 nm (I_590_/I_525_) exhibited a good linear correlation (R^2^ = 0.9964) with the Cys concentrations in range of 0–40 μM with the detection limit was 0.48 μM (Figure S2). In addition, the fluorescence intensity ratio at 590 and 525 nm (I_590_/I_525_) increased from 0.64 to 5.19, which corresponded to an ~8-foldenhancement. Finally, the fluorescence intensity ratio at 590 and 525 nm (I_590_/I_525_) gradually increased to a plateau within 180 s (3 min) in the presence of Cys (Figure 1d).

### 2.3. The Optical pH Range and Selectivity of Probe ***1*** to Cys

**Figure 2:**
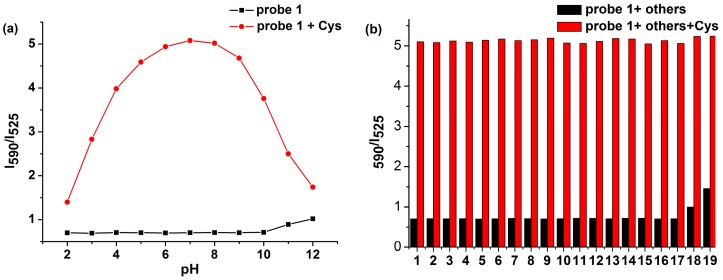
(**a**) Effect of pH on the fluorescence intensity ratio(I_590_/I_525_) of probe **1** in the absence of Cys and in the presence of Cys (50 μM); (**b**) Fluorescence intensity ratio (I_590_/I_525_) of probe **1** for various analytes (1–19 represent: 1. Al^3+^, 2. Cu^2+^, 3. Fe^3+^, 4. NO_3_^−^, 5. NO_2_^−^, 6. SO_4_^2−^, 7. SO_3_^2−^, 8. S_2_O_3_^2−^, 9. F^−^, 10. Cl^−^, 11. Leu, 12. Tyr, 13. Arg, 14. Glu, 15. Lys, 16. Thr, 17. Ser, 18. GSH, 19. Hcy). Each spectrum was collected after 5 min of mixing each analyte with probe **1** in DMSO/PBS solution (1/1, *v*/*v*, pH = 7.4, 10 mM) at 37 °C. λ_ex_ = 410 nm, d_ex_ = 3nm, d_em_ = 5 nm.

In addition, the pH effect on the probe was also evaluated. These experiments were carried out at a pH range from 2 to 12 with the concentration of probe **1** (10 μM) and Cys (50 μM). Experimental results showed that the fluorescence intensity of probe **1** itself was unaffected over a wide pH range, which indicated that the probe was quite stable (Figure 2a).However, while in the presence of Cys, the ratio value (I_590_/I_525_) was enhanced greatly in the pH range from 6 to 8 and exhibited relatively weak change in the pH ranges (<6 and >8). The probable explanation is the change in the protonation state, which affects the reactivity of Cys at different pH. All results indicated that probe **1** hada good fluorescence response toward Cys in the neutral pH range.

The selectivity of probe **1** (10 μm) for Cys over Hcy, GHS, and other amino acids, such as leucine (Leu), tyrosine (Tyr), argnine (Arg), glutamic acid (Glu), lysine (Lys), threonine (Thr), and serine (Ser), and various ions, such as Al^3+^, Cu^2+^, Fe^3+^, NO_3_^−^, NO_2_^−^, SO_4_^2−^, SO_3_^2−^, S_2_O_3_^2−^, F^2−^, Cl^−^, were also investigated. Remarkable fluorescent enhancement could be observed upon the addition of Cys; the presence of other analytes in probe **1** solution induced small or no fluorescence change at 590 nm, even if the training time was up to 60 min (Figure S3). Subsequently, the detections of Cys with probe **1** in the presence of these amino acids and ions were also effective, indicating that probe **1** was highly selective for Cys over Hcy, GHS and other analytes (Figure 2b). Probe **1** showed small changes at millimolar level of GSH, which is important for Cys detection in living systems, because the abundant GSH in living cells could be the main source of interference during detection of Cys. All these results demonstrated that probe **1** had high selectivity for Cys.

### 2.4. Proposed Mechanism of Probe ***1*** for Cys

**Scheme 2:**
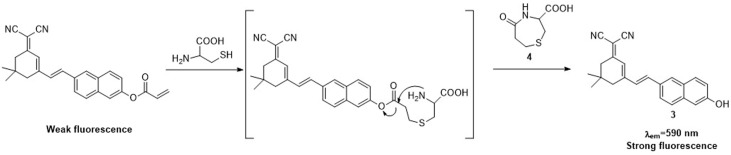
Proposed mechanismof probe **1** for Cys.

The acrylate moiety would conjugate addition with Cys to generate thioethers and then intramolecular cyclization to yield compound **3** and compound **4** (Scheme 2). To verify the above proposed reaction mechanism, fluorescence spectroscopic, MS, and HPLC studies were carried out by adding excess Cys to a solution of probe **1**. Firstly, the spectra of compound **3** were consistent with probe **1** after treatment with Cys, suggesting that compound **3** was the product (Figure S4). Furthermore, the peak at 176.06 and 341.03 corresponding to **3** and **4** were clearly observed during the mass spectrum of the reaction mixture (Figure S5). This clearly indicated that compounds **3** and **4** were formed in the reaction. Finally, the HPLC analysis of the reaction mixture showed that a new peak with the same retention time (6.98 min) as the reference compound **3** (Figure S6), which confirmed that the reaction of probe **1** with Cys produced fluorophore compound **3**. These data were in good agreement with the proposed mechanism. The high selectivity of probe **1** for Cys over Hcy and GSH can be explained by the fact that probe **1** had much faster kinetic rate of the intramolecular cyclization reactions for Cys.

### 2.5. Cellular Imaging

**Figure 3:**
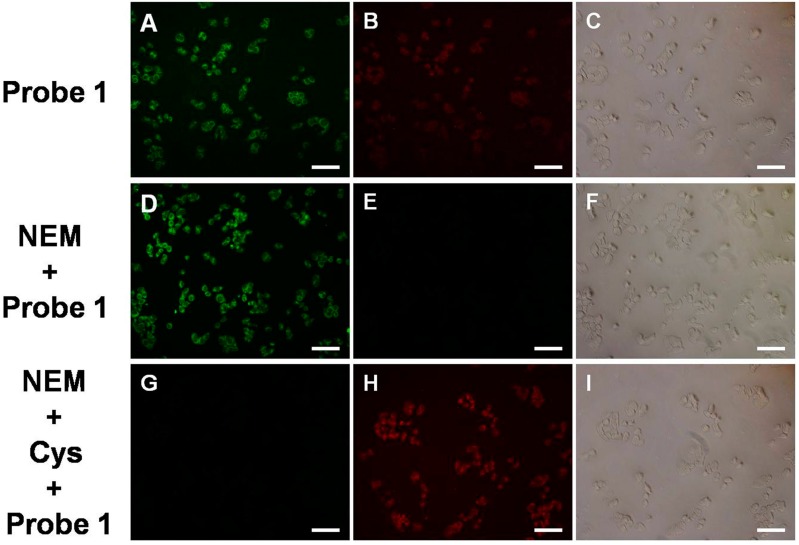
Bright-field and fluorescence images of HeLa cells stained with the probe **1**: (**A**–**C**) bright-field and fluorescence images of cells incubated only with the probe (10 μM) for 20 min; (**D**–**F**) bright-field and fluorescence images of cells incubated with NEM (100 μM) for 30 min, and then treated with the probe (10 μM) for 20 min; (**G**–**I**) bright-field and fluorescence images of the cells incubated with NEM (100 μM) for 30 min, then incubated with Cys (50 μM) for 20 min and incubated with probe **1** (10 μM) for last 20 min. Scale bar, 200 μm.

Inspired by the above results, the potential application of probe **1** to Cys in live cell imaging was examined. Firstly, the cell viability indicated that the probe had low toxicity and superior bio-compatibility towards cultured cell lines (Figure S7). When HeLa cells were incubated with probe **1**, strong and weak fluorescence was observed in the red channel and green channel respectively. As a control experiment, when the cells were pretreated with *N*-ethylmaleimide (NEM, a thiol blocking agent, 100 μM) and then incubated with probe **1** for 20 min, they showed weak fluorescence in the green channel and almost no fluorescence signal in the red channel, resulting in a distinct enhancement of emission ratio (Green/Red) in the stained cells (Figure S8). Moreover, when the cells were firstly incubated with NEM, then incubated with 50 μM of Cys for 20 min and finally incubated with probe **1** for the last 20 min, it was found that these cells displayed marked fluorescence enhancement in the red channel and decrease in the green channel (Figure 3). The quantified data of the ratio (Green/Red) of fluorescence intensity showed remarkable decrease. These results suggest that probe **1** has great potential for biological applications.

## 3. Materials and Methods

### 3.1. Materials and Apparatus

All chemical reagents and solvents were purchased from commercial suppliers and used without further purification. Buffers for optical studies were prepared with ultrapure water. TLC analysis was carried out on silica gel plates (GF-254). ^1^H- and ^13^C-NMR spectra were obtained on Bruker Avance III 500 MHz spectrometer (Bruker, Karlsruhe, Germany). MS spectra were obtained on a Thermo Scientific DSQII GC/MS spectrometer (Thermo Fisher Scientific, Waltham, MA, USA). HRMS spectra were obtained on a Bruker ApexII by means of the ESI technique (Bruker, Karlsruhe, Germany). Absorption spectra were obtained on a Lambda 35 UV-vis spectrophotometer (Perkin Elmer, Waltham, MA, USA). Fluorescence spectra were obtained on aShimadzuRF-5301PC Fluorescence Spectrometer (Shimadzu, Tokyo, Japan). All titrations were carried out in PBS/DMSO solution (1:1, *v*/*v*, pH = 7.4).

### 3.2. Synthesis of 2-(3,5,5-Trimethylcyclohex-2-en-1-ylidene)malononitrile (***2***)

Isophorone (6.91 g, 0.05 mol) and malononitrile (3.96 g, 0.06 mol) were dissolved in absolute ethanol (100 mL), followed by addition of piperidine (0.25 mL, 2.5 mmol). Then the mixture was refluxed for 6 h under nitrogen atmosphere. After the solvent was added to ice water, the precipitation was dissolved in dichloromethane, washed with water, and dried over MgSO_4_. Finally, the solvent was evaporated under reduced pressure and the crude product was purified by silica column chromatography (petroleum/ethyl acetate = 3:1, *v*/*v*) to give compound **2** (7.78 g, 83% yield). m.p.: 71–72 °C, ^1^H-NMR (500 MHz, CDCl_3_) δ6.62 (1H, d, *J* = 1.1 Hz), 2.51 (2H, s), 2.17 (2H, s), 2.03 (3H, s), 1.01 (6H, s) ppm; ^13^C-NMR (126 MHz, CDCl_3_) δ 170.93, 160.08, 119.55, 111.08, 77.10, 44.65, 41.65, 33.55, 26.83, 24.35 ppm. HRMS [M + H]^+^: Calcd. for C_12_H_14_N_2_ 186.1157, Found 187.1230.

### 3.3. Synthesis of (E)-2-(3-(2-(6-Hydroxynaphthalen-2-yl)vinyl)-5,5-dimethylcyclohex-2-en-1-ylidene)Malononitrile (***3***)

Compound **2** (1.86 g, 0.1 mol) and 6-Hydroxy-2-naphthaldehyde (1.72 g, 0.01 mol) were dissolved in ethanol (40 mL), followed by five drops of piperidine. The mixture was refluxed overnight under a nitrogen atmosphere until TLC indicated the end of the reaction. After cooling to room temperature, red precipitate was produced, and then filtered to give compound **3** (2.93 g, 86% yield). m.p.: 223–224 °C, ^1^H-NMR (500 MHz, DMSO) δ 10.02 (1H, s), 8.01 (1H, s), 7.77 (3H, m, *J* = 5.1 Hz), 7.39 (2H, m, *J* = 16.1 Hz), 7.21 (2H, m), 6.86 (1H, s), 2.56 (2H, s), 2.53 (2H, s), 1.01 (6H, s); ^13^C-NMR (126 MHz, DMSO) δ 169.63, 156.34, 155.58, 137.64, 134.80, 130.04, 129.67, 128.64, 127.80, 126.92, 126.23, 123.72, 121.49, 118.93, 113.29 112.66, 109.16, 75.11, 41.74, 37.64, 32.72, 26.63 ppm. The purity of compound **3** was 99.8%, which was determined by HPLC analysis using a VP-ODS column, H_2_O/EtOH = 15:85, 1 mL/min, retention time = 6.984 min (Figure S10). HRMS [M + H]^+^: Calcd. for C_23_H_20_N_2_O340.1576, Found 341.1648.

### 3.4. Synthesis of (E)-6-(2-(3-(Dicyanomethylene)-5,5-dimethylcyclohex-1-en-1-yl)vinyl)naphthalen-2-yl Acrylate (***1***)

Compound **3** (0.34 g, 0.001 mol) and triethylamine (0.5 mL) were dissolved in dichloromethane (20 mL) at 0 °C. A solution of acryloyl chloride (0.27 g, 0.003 mol) in 10 mL dichloromethane was added to the mixture in 30 min. The mixture was stirred at 0 °C for 1 h, and then stirred at room temperature for 2 h. The organic phase was washed with water, and dried over MgSO_4_, then filtered and concentrated under reduced pressure. The crude solid was purified by silica column chromatography (dichloromethane/methanol = 20:1, *v*/*v*) to give probe **1** (0.24 g, 61% yield). m.p.: 197–198 °C, ^1^H-NMR (500 MHz, DMSO) δ 8.15 (1H, s), 7.92 (3H, m, *J* = 8.9 Hz), 7.71 (1H, d, *J* = 2.1 Hz), 7.51 (1H, d, *J* = 16.1 Hz), 7.45 (2H, m), 6.89 (1H, s), 6.56 (1H, d, *J* =10.0 Hz), 6.43 (1H, m, *J* =10.3 Hz), 6.16 (1H, d, *J* = 10.3 Hz), 2.56 (4H, d, *J* = 18.2 Hz), 0.99 (6H, s); ^13^C-NMR (126 MHz, DMSO) δ = 169.74, 163.74, 155.16, 148.23, 136.76, 134.17, 130.59, 129.52, 128.75, 124.29, 122.51, 121.68, 118.14, 112.91, 76.02, 41.77, 37.66, 31.16, 26.93. The purity of probe **1** was 96.0%, which was determined by HPLC analysis using a VP-ODS column, H_2_O/EtOH = 15:85, 1 mL/min, retention time = 10.874 min (Figure S9). HRMS [M + H]^+^: Calcd. for C_26_H_22_N_2_O_2_394.1681, Found 395.1754.

### 3.5. Absorption and Fluorescence Spectroscopy

Stock solution of probe **1** (2.0 × 10^−3^ M) was prepared in DMSO. Stock solutions (1 mM) of the analytes including: Cys, Hcy, GHS, Al^3+^, Cu^2+^, Fe^3+^, NO_3_^−^, NO_2_^−^, SO_4_^2−^, SO_3_^2−^, S_2_O_3_^2−^, F^−^, Cl^−^, Leu, Tyr, Arg, Glu, Lys, Thr, Ser were prepared in ultrapure water. For a typical optical study, the probe **1** (10 μM) solution in PBS/DMSO solution (1:1, *v*/*v*, pH = 7.4, 10 mM) was prepared. Then 3.0 mL of the solution was placed in a quartz cuvette at room temperature. For fluorescence measurements, slit width was set at d_ex_ = 3 nm, d_em_ = 5 nm.

### 3.6. Cell Incubation and Imaging

HeLa cells were provided by the Chinese Academy of Science. HeLa cells were cultured in Dulbecco’s modified Eagle’s medium (DMEM) supplemented with 10% fetal bovine serum (FBS) at 37 °C under an atmosphere of 5% CO_2_. The images of cells were visualized and photographed by a fluorescence microscope (Nikon, Tokyo, Japan). In the experiment of cell imaging, cells were incubated with 10 μM of probe **1** for 20 min at 37 °C and washed three times with pre-warmed PBS, and then imaged. For *N*-ethylmaleimide (NEM, a thiol blocking agent)-treated experiments, the HeLa cells were pretreated with 500 μM NEM at 37 °C for 30 min, washed with PBS for three times, then incubated with 10 mM probe **1** at 37 °C for 20 min. Moreover, the cells were firstly incubated with NEM, then incubated with 50 μM of Cys for 20 min and finally incubated with probe **1** for the last 20 min. Cell imaging was then carried out after washing cells with pre-warmed PBS buffer in each case.

## 4. Conclusions

In summary, we have developed a novel ratiometric probe for detection of Cys. This probe can be prepared in three steps with an overall yield of 44%. Upon the addition of Cys, a large fluorescence enhancement was observed within 180 s, and the detection limit was 0.48 μM. Importantly, this probe showed a selective detection process for Cys with distinct turn-on signal changes over various analytes, including the similarly structured Hcy and GSH. In addition, this probe had low cytotoxicity, and could be applied to imaging intracellular Cys with fluorescence enhancement. Overall, this probe can be used as a new valuable research tool for Cys detection.

## Supplementary Materials

The following are available online.
