# Supplementary material for: A Novel Dicyanoisophorone-Based Ratiometric Fluorescent Probe for Selective Detection of Cysteine and Its Bioimaging Application in Living Cells
Source: Molecules. 2018 Feb 22;23(2):475. doi: 10.3390/molecules23020475 (PMC6017397; doi:10.3390/molecules23020475)
Supplement: Supplementary file 1 [file molecules-23-00475-s001.docx]

Supplementary Data

for

**A novel dicyanoisophorone-based ratiometric fluorescent probe for selective detection of cysteine and its bioimaging application in living cells**

Hengrui Zhang, Nan Qin, Zhijie Fang*

School of Chemical Engineering, Nanjing University of Science & Technology,

200 Xiao Ling Wei, Nanjing 210094, P.R. China

*Corresponding author. E-mail: [zjfang@njust.edu.cn](mailto:zjfang@niust.edu.cn)

**Table of contents:**

**1. Determination of the detection limit…………………………………Page S2**

| **2. Structure characterizations of compound 3, 2 and probe 1……….Page S3-5** |
| --- |
| **3. Additional data…………………………………………………….....Page S6-8** |
| 1. **Determination of the detection limit**  The detection limit of probe **1** was determined from the following equation: $SD=K\text{*}\frac{SD}{S}$   Where K = 3; SD is the standard deviation of the blank solution; S is the slope of the calibration curve. |

**2. Structure characterizations of compound 2, 3 and probe 1**


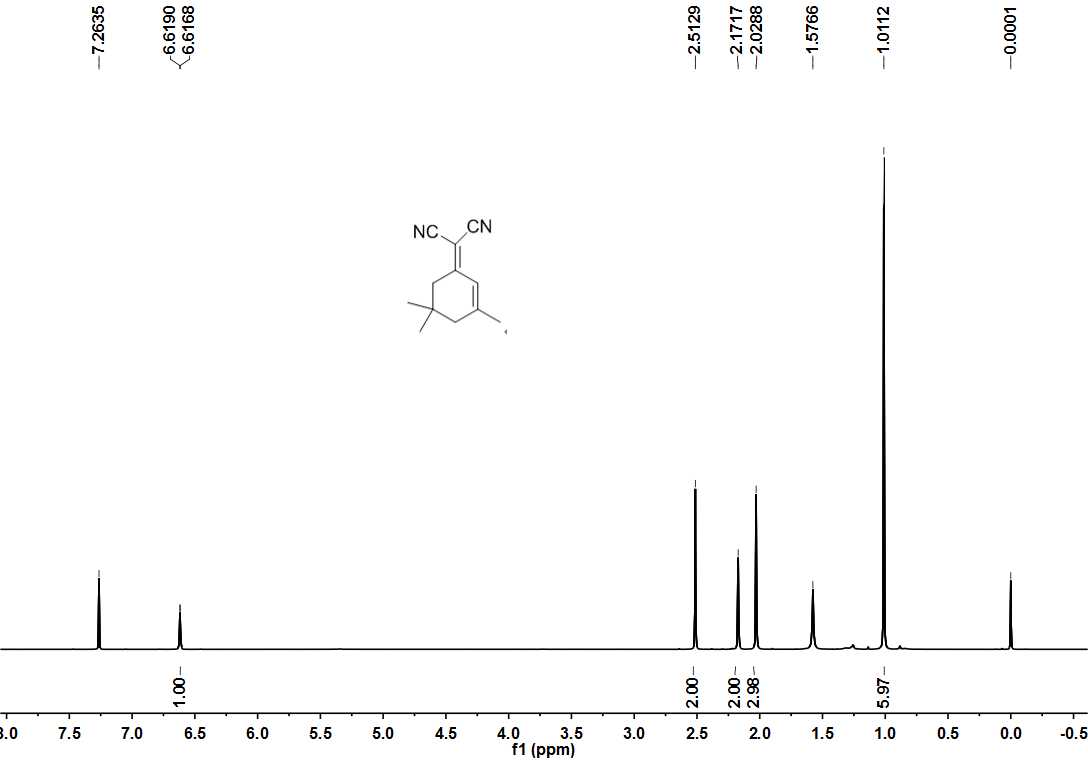


^1^H-NMR spectrum of compound **2**

**
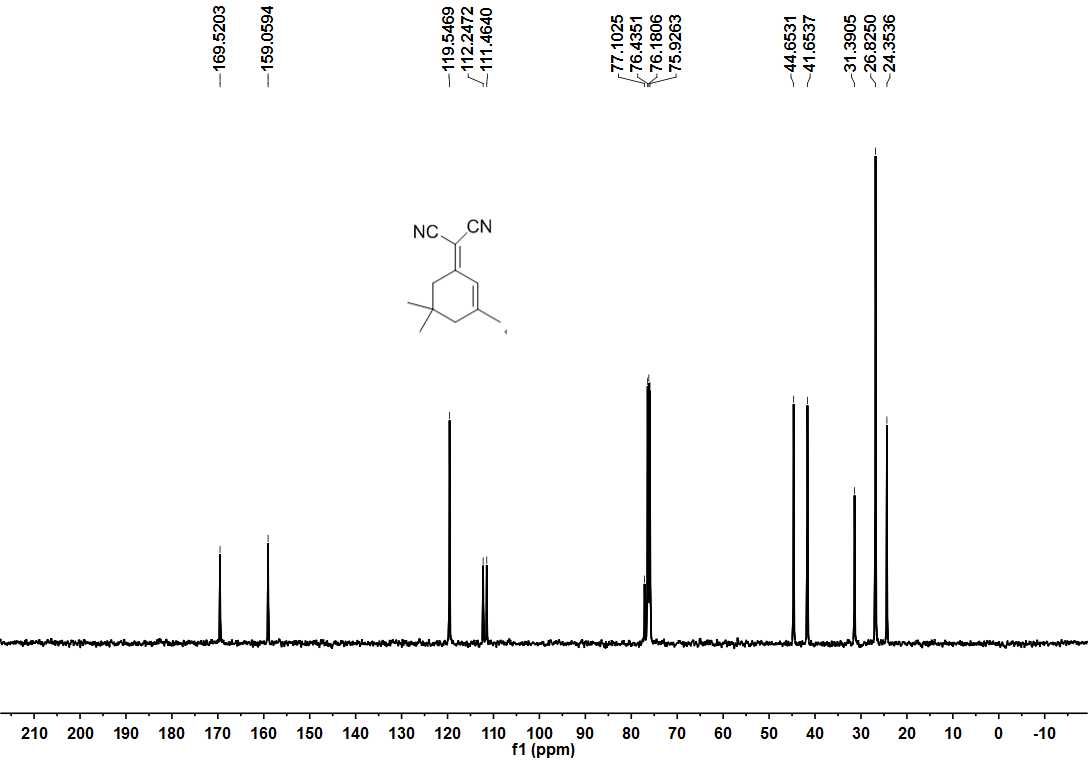
**

^13^C-NMR spectrum of compound **2**


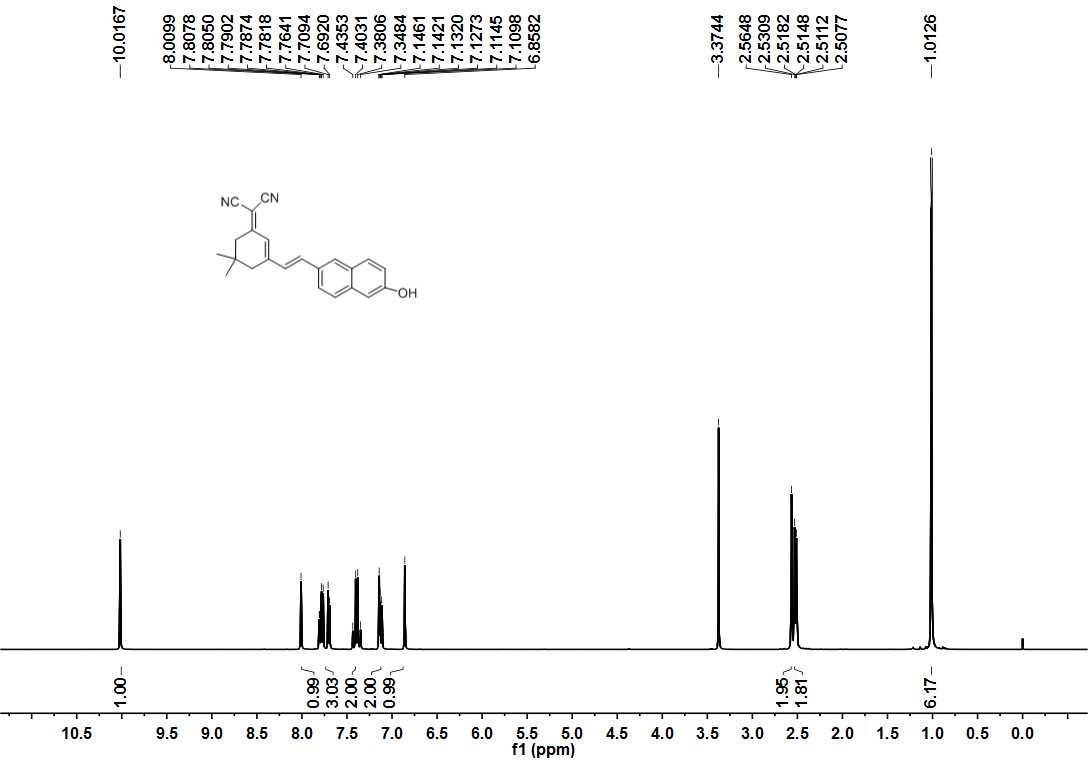


^1^H-NMR spectrum of compound **3**


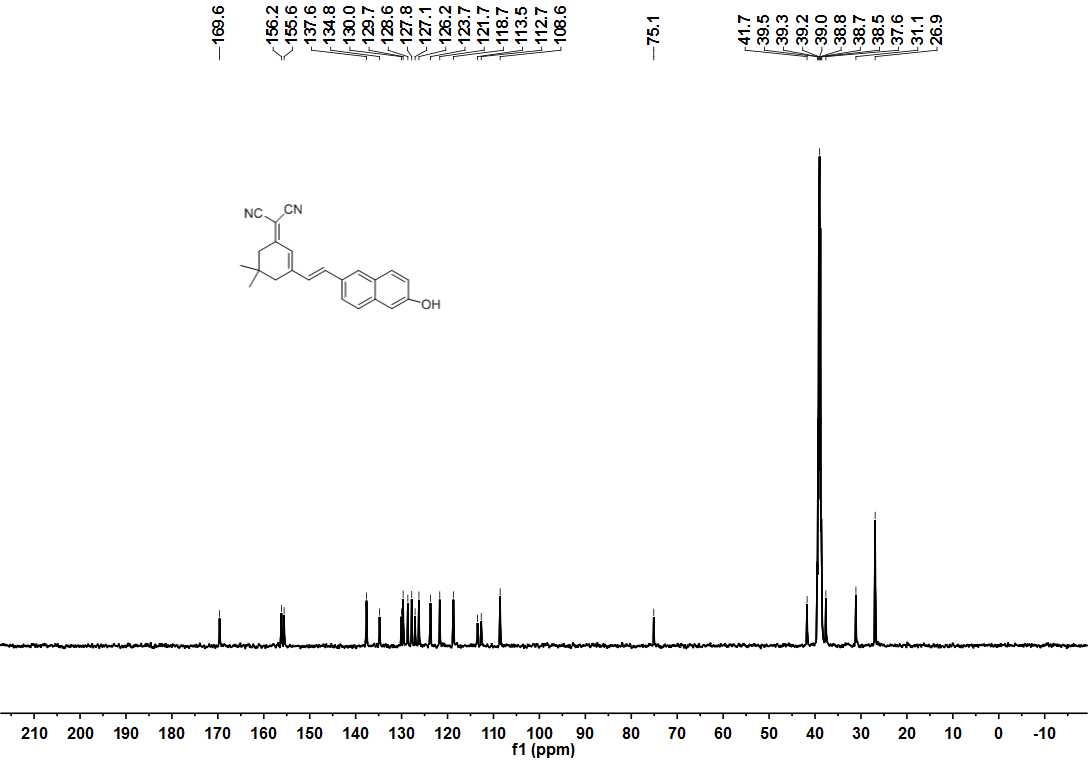


^13^C-NMR spectrum of compound **3**


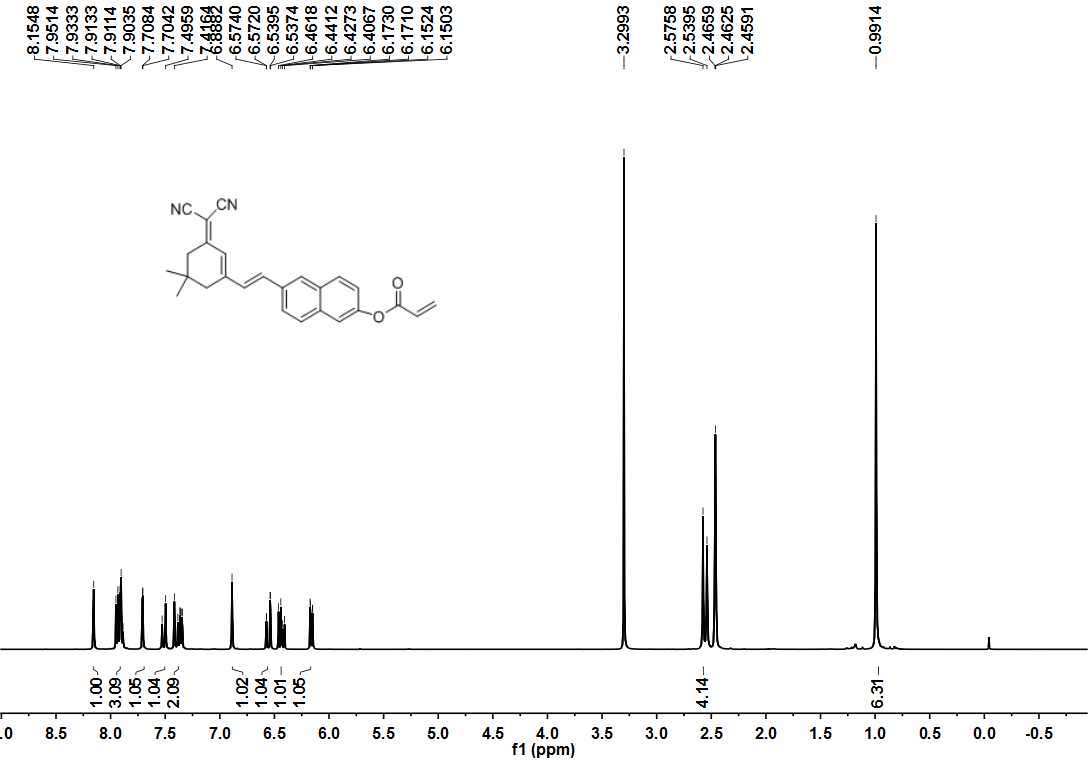


^1^H-NMR spectrum of probe **1**


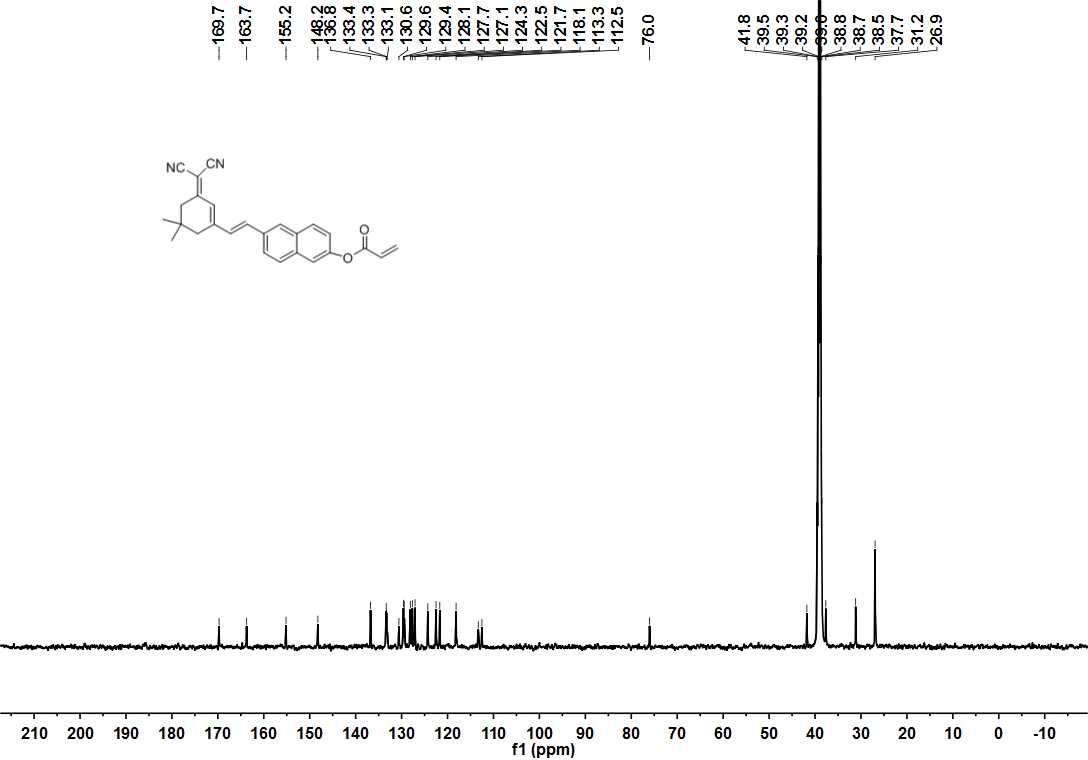


^13^C-NMR spectrum of probe **1**

1. **Additional data**


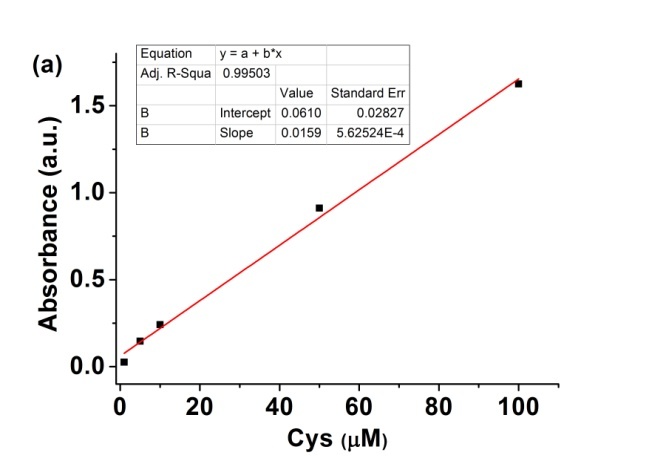

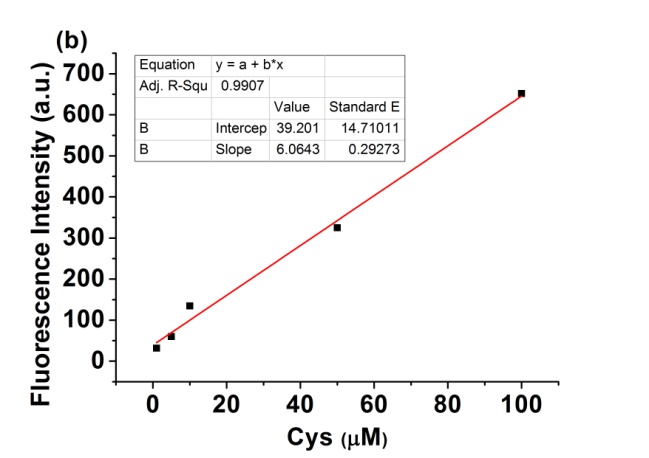


**Figure S1**. The linearity of absorbance (a) and fluorescence (b) against different probe concentration(1μM, 5μM, 10μM, 50μM, 100μM).


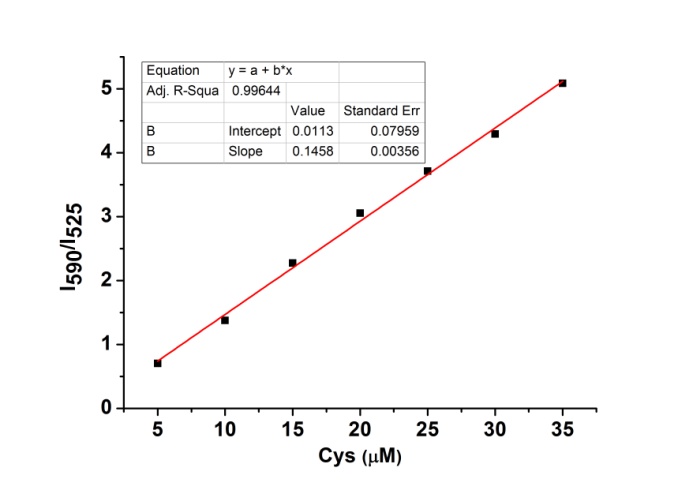


**Figure S2**. The linear fitting curve between the fluorescence intensity ratio (I_590_/I_525_) and the concentration of Cys.

**Figure S3**. Fluorescence intensity ratio (I_590_/I_525_) of probe **1** for various analytes (1-20 represent: 1. Al^3+^, 2. Cu^2+^, 3. Fe^3+^, 4. NO_3_^-^, 5. NO_2_^-^, 6. SO_4_^2-^, 7. SO_3_^2-^, 8. S_2_O_3_^2-^, 9. F^-^, 10. Cl^-^, 11. Leu, 12. Tyr, 13. Arg, 14. Glu, 15. Lys, 16. Thr, 17. Ser, 18. GSH, 19. Hcy, 20. Cys).


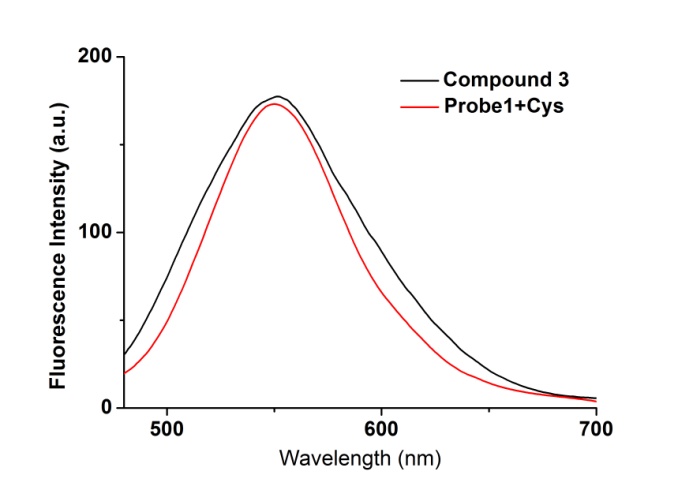


**Figure S4**. Fluorescence spectra of compound **3** (10 µM) and probe **1** (10 µM with 50 µM of Cys) in PBS/DMSO solution (1:1, v/v, pH = 7.4, 10 mM). Spectra of probe **1** with Cys were recorded 5 min after exposure at 37°C.

**Figure S5**. MS spectrum of the products from the reaction of probe **1** (10 µM, DMSO/PBS, 1:1, v/v, pH 7.4, 10 mM) with 50μM of Cys. Spectrum was obtained 20 min after exposure at 37°C.

**Fig. S6.** Data of HPLC analysis (column: Shim-pack VP-ODS, 250×4.6 mm, 5 μm, eluent: CH_3_OH-H_2_O 85:15, v/v, flow rate: 1 mL/min, temperature: 25°C, detection wavelength: 445 nm). The HPLC traces are: (a) the probe **1**, (b) the reaction sample of probe **1** (10 μM) with Cys (20 μM), (c) the reaction sample of probe **1** (10 μM) with Cys (50 μM), (d) the reference sample of compound **3**.


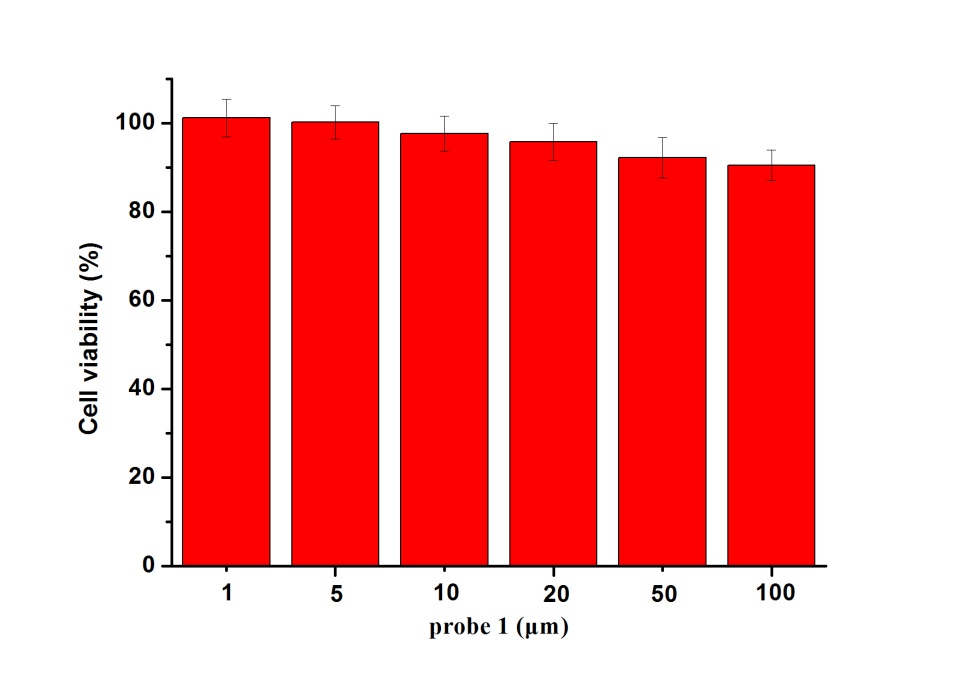


**Fig. S7.**Viable HeLa cells after treatment with different concentrations of probe **1**. The cell viability was observed via MTT assay. HeLa cells were cultured for 4h in the presence of probe **1.**

**Fig. S8.** (a) Average fluorescence intensities of images (b) the average ratio of green/red fluorescence intensity at three fields of view. Fluorescence intensity quantitation was analyzed using the Image J. The results were presented as mean ± SE with replicates n = 3

**
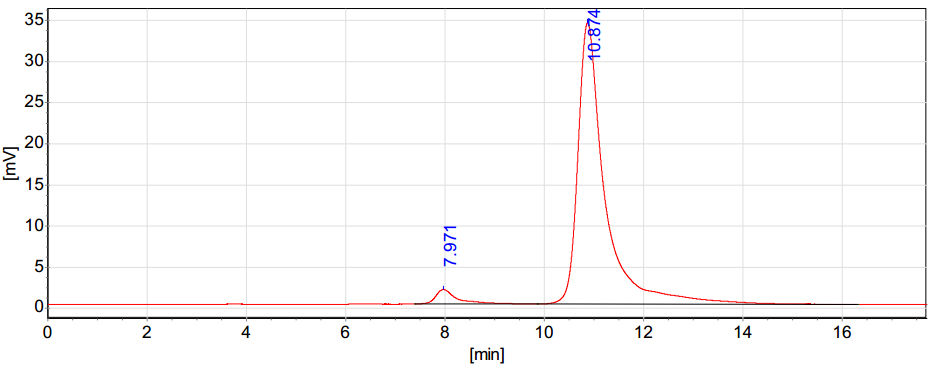
**

Detector A 410 nm

| Peak | Ret. time | Area | Height | Conc.% |
| --- | --- | --- | --- | --- |
| 1 | 7.971 | 54241 | 1766 | 4.03316 |
| 2 | 10.874 | 1290633 | 34188 | 95.96684 |
| Total |  | 1344874 | 35954 |  |

**Fig. S9.** The purity of probe **1:** VP-ODS column, H_2_O/EtOH = 15:85, 1 ml/min, retention time=10.874 min.


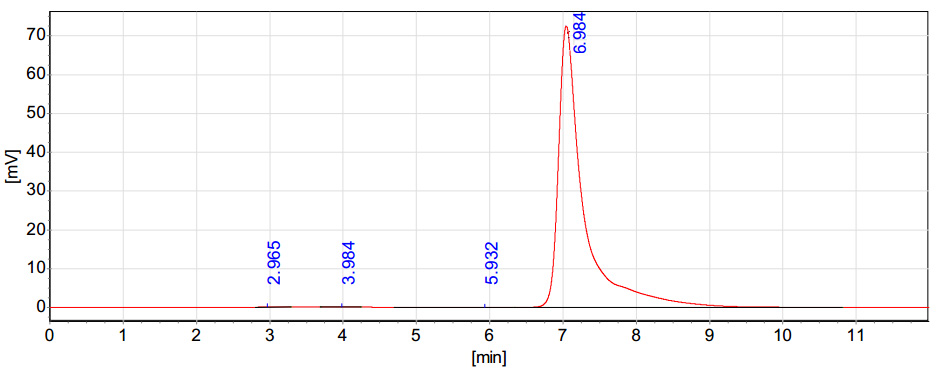


Detector A 410 nm

| Peak | Ret. time | Area | Height | Conc.% |
| --- | --- | --- | --- | --- |
| 1 | 2.965 | 1546 | 116 | 0.09029 |
| 2 | 3.984 | 313 | 23 | 0.01828 |
| 3 | 5.932 | 705 | 15 | 0.04113 |
| 4 | 6.984 | 1711849 | 72584 | 99.85030 |
| Total |  | 1714416 | 72738 |  |

**Fig. S10.** The purity of compound **3:** VP-ODS column, H_2_O/EtOH = 15:85, 1 ml/min, retention time=6.984 min.
